# Shoulder strength, scapular displacement, and supraspinatus morphology in male and female competitive water polo players: a cross-sectional study

**DOI:** 10.3389/fspor.2026.1933053

**Published:** 2026-10-14

**Authors:** José Ángel del-Blanco-Muñiz, Alberto Bermejo-Franco, María-José Giménez, Federico Salniccia, Daniel Mendoza-Castejón, Alfonso Trinidad

**Affiliations:** 1 Department of Physiotherapy, Faculty of Medicine, Health and Sports, Universidad Europea de Madrid, Madrid, Spain; 2 Department of Sport Sciences, Faculty of Medicine, Health and Sports, Universidad Europea de Madrid, Madrid, Spain; 3 Department of Education and Educational Innovation, Faculty of Law, Education and Humanities, Universidad Europea de Madrid, Madrid, Spain

**Keywords:** injury prevention, rotator strength, sex differences, shoulder biomechanics, water polo

## Abstract

**Background:**

Water polo is an Olympic team sport played by both men and women that combines repetitive throwing and aquatic propulsion, placing unique biomechanical demands on the shoulder. However, sex-related differences in shoulder function and structure remain poorly understood. This study compared the functional and structural shoulder profiles of male and female competitive water polo players.

**Methods:**

A cross-sectional study included 52 competitive water polo players (26 men and 26 women; 18–30 years). Internal and external rotator strength was assessed using a handheld dynamometer, and the internal-to-external rotation strength ratio (IR/ER ratio) was calculated. Scapular position and displacement were evaluated using a PALM® device, and supraspinatus tendon thickness was measured by ultrasound. Univariate analyses were followed by multiple linear regression adjusted for height and body mass.

**Results:**

Men showed greater internal rotation strength (*p* = 0.011) and a higher IR/ER ratio (*p* = 0.004), whereas women exhibited greater scapular displacement (*p* = 0.004). No significant sex differences were observed in external rotation strength (*p* = 0.052), supraspinatus tendon thickness (*p* = 0.384), or arm dominance (*p* > 0.05). After adjustment, sex was no longer associated with internal rotation strength or the IR/ER ratio. However, greater scapular displacement in women remained independently associated with sex (*p* = 0.005; adjusted *p* = 0.020).

**Conclusions:**

Sex differences in shoulder strength were largely explained by anthropometric characteristics. In contrast, greater scapular displacement in female players remained independent of body size, suggesting a sex-specific biomechanical adaptation that may be relevant for shoulder injury prevention. These findings contribute to a better understanding of sex-specific shoulder adaptations in water polo and may support the development of more individualized assessment and injury prevention strategies.

## Introduction

Overhead sports such as volleyball, baseball, handball, and tennis are characterized by sport-specific movement patterns and loading demands that may lead to shoulder adaptations and injuries (1). Recent studies indicate a notably high incidence of shoulder injuries, with up to 49% of overhead athletes reporting shoulder pain and prevalence rates between 5% and 36% across different sports and levels of play (2–4). In particular, in water polo players, shoulder injuries accounted for 25% of all self-reported injuries and 16% of physiotherapist-reported injuries, making it the most frequently injured anatomical site and accounting for 25% of all lost training days (5).

This issue is particularly relevant in water polo, one of the aquatic disciplines recognized by World Aquatics. Since its origins in the mid-nineteenth century (6), water polo has distinguished itself from other aquatic sports, such as swimming, diving, and artistic swimming, through its unique combination of technical, tactical, and physical demands (7). Unlike other aquatic sports, water polo combines repeated high-intensity swimming with complex throwing mechanics, physical contact, and vertical treading positions, making it one of the most physically demanding team sports (8–10). The sport requires explosive upper-limb activity, rapid directional changes, and sustained overhead motions, which place significant load on the shoulder complex and trunk musculature (11, 12). Consequently, the shoulder represents the anatomical region most frequently affected by injury in water polo (9, 13).

Despite increasing research on water polo injuries and shoulder-related disorders, sex-specific evidence remains limited, highlighting the need for studies comparing shoulder characteristics between men and women (8). Evidence from competitive swimming supports the potential relevance of sex differences, with female swimmers showing shorter arm strokes, a higher number of arm revolutions, and a greater incidence of injuries across several body regions compared with males (13). These differences may be related to intrinsic factors such as joint laxity, neuromuscular control, and relative strength, which could influence injury patterns and mechanisms (13).

In water polo, Miller et al. (2018) reported significantly higher shoulder injury rates in female players, who exhibited nearly 2.4 times the rate observed in males (8.09 vs. 3.40 injuries per 100 participant-years). Potential explanations include greater shoulder laxity, reduced rotator cuff strength, and altered scapular control in females (9). However, evidence regarding overall injury prevalence remains inconsistent, with higher rates reported in males in some populations and in females among collegiate players (8). These findings suggest that sex-related differences in overall injury risk may vary according to the study population and competition level.

Additional distinctions are observed in injury severity. Mountjoy et al. (2019) found that although the type, location, and causes of injuries were similar between sexes, male players experienced significantly more time-loss injuries, particularly during match play. Among the most severe injuries, defined as those resulting in an absence of ≥3 weeks, 80% occurred in male athletes. These findings may reflect differences in matching intensity, style of play, or injury reporting behaviors between sexes (14). Although previous studies have reported sex-related differences in shoulder injury rates, overall injury prevalence, and injury severity among water polo players, these findings examine different epidemiological outcomes and provide limited information about the mechanisms underlying such differences. Consequently, the relationship between sex and shoulder injury risk in water polo remains incompletely understood.

Shoulder injury risk in overhead athletes is multifactorial, involving non-modifiable factors such as sex, age, previous injury, and playing exposure, as well as modifiable functional and structural factors, including rotator cuff strength, internal-to-external rotation strength balance, scapular displacement, and tendon adaptations (1, 15). The internal-to-external rotation strength ratio characterizes the relative strength relationship between the agonist and antagonist muscle groups and may provide complementary information to absolute strength values when assessing shoulder muscle balance.

Although sex-related differences in shoulder characteristics have been reported in overhead athletes (16), evidence in water polo players is limited. Therefore, evaluating these parameters may help identify sex-specific shoulder adaptations and provide insight into potential mechanisms associated with injury risk. Despite the growing body of research on shoulder function in overhead athletes, most studies have focused on land-based sports or swimming populations, limiting the applicability of their findings to water polo. The unique biomechanical demands of water polo, combining repetitive overhead throwing, aquatic propulsion, physical contact, and unstable body positioning, may lead to distinct functional and structural shoulder adaptations.

Based on previous evidence from overhead sports and the reported sex-related differences in injury patterns among water polo players, we hypothesized that male and female water polo players would exhibit different functional and structural shoulder profiles, including differences in rotator cuff strength, internal-to-external rotation strength balance, scapular displacement, and supraspinatus tendon thickness.

Therefore, the present study aims to provide a comprehensive, sport-specific analysis of shoulder function in male and female water polo players, integrating functional (strength and internal-to-external rotator strength ratio), biomechanical (scapular position and displacement), and structural (supraspinatus tendon thickness) variables. By directly comparing these characteristics between sexes, this study seeks to contribute to a better understanding of sex-specific shoulder adaptations in competitive water polo and to provide information that may support more targeted injury prevention strategies.

## Methodology

### Study design and setting, ethical considerations

This was a cross-sectional, observational study designed to analyze sex-based differences in shoulder functional and structural variables among competitive water polo players. Data collection was conducted at the facilities of Universidad Europea de Madrid between December 2024, and October 2025.

The study was carried out in accordance with the ethical principles of the Declaration of Helsinki and was approved by the Ethics Committee of the European University of Madrid (Code: CIPI/20/007). The methodological design followed the STROBE (Strengthening the Reporting of Observational Studies in Epidemiology) guidelines for observational studies (17).

### Participants

Participants were informed about the study's purpose, procedures, and objectives. Participation was voluntary, and all athletes provided written informed consent prior to inclusion. A total of 52 water polo players (26 men and 26 women) were included in the study. Participants were classified according to the Participant Classification Framework proposed by McKay et al. (2022), based on their training volume and competitive level (16). All athletes trained at least four to five days per week and regularly competed in organized regional or national tournaments. Participants were recruited through non-probability convenience sampling from two federated water polo clubs, comprising one men's team and one women's team. Data were collected during the competitive season over an approximately 9-month period. Assessments were scheduled according to the availability of the participating teams. All participants were field players; no goalkeepers were included in the study.

Athletes were eligible if they were between 18 and 30 years old, had at least three years of continuous water polo practice, trained a minimum of four to five days per week, and were actively competing in regional or national tournaments. To avoid bias in functional outcomes, participants were required to be free of shoulder injuries during the six months prior to data collection.

Athletes were excluded if they had a history of shoulder surgery, current shoulder pain, or any musculoskeletal condition that could interfere with the assessment procedures or compromise the accuracy of the measurements. Athletes with current shoulder pain were excluded to minimize the potential influence of pain-related inhibition or compensatory movement patterns on strength, scapular positioning, and tendon morphology assessments. Before enrolment, all participants received detailed written information about the study and provided written informed consent.

### Sample size

A total of 66 athletes were initially invited to participate (34 men and 32 women). Of these, 52 water polo players met the inclusion criteria and agreed to participate, resulting in a response rate of 78.8%. All collected data were reviewed to verify completeness and compliance with the eligibility criteria, and no cases were excluded due to missing data.

No formal *a priori* sample size calculation was performed because this was an exploratory cross-sectional study based on a convenience sample of competitive water polo players recruited from two federated clubs. The final sample was therefore determined by the number of eligible athletes available during the recruitment period. An a posteriori sample size estimation indicated that, assuming a two-sided comparison between two independent groups, an alpha level of 0.05% and 80% statistical power, approximately 26 participants per group would be required to detect a large, standardized effect size (Cohen's d = 0.79). Therefore, the final sample of 52 participants was considered adequate to detect large between-group differences, although smaller effects may not have been identified.

Although the study was based on a convenience sample, the final sample size is comparable to previous studies assessing shoulder function in overhead athletes and is considered adequate for exploratory sex-comparison analyses (18–20). Figure 1 presents the flow diagram of the study, illustrating the recruitment and selection process.

**Figure 1:**
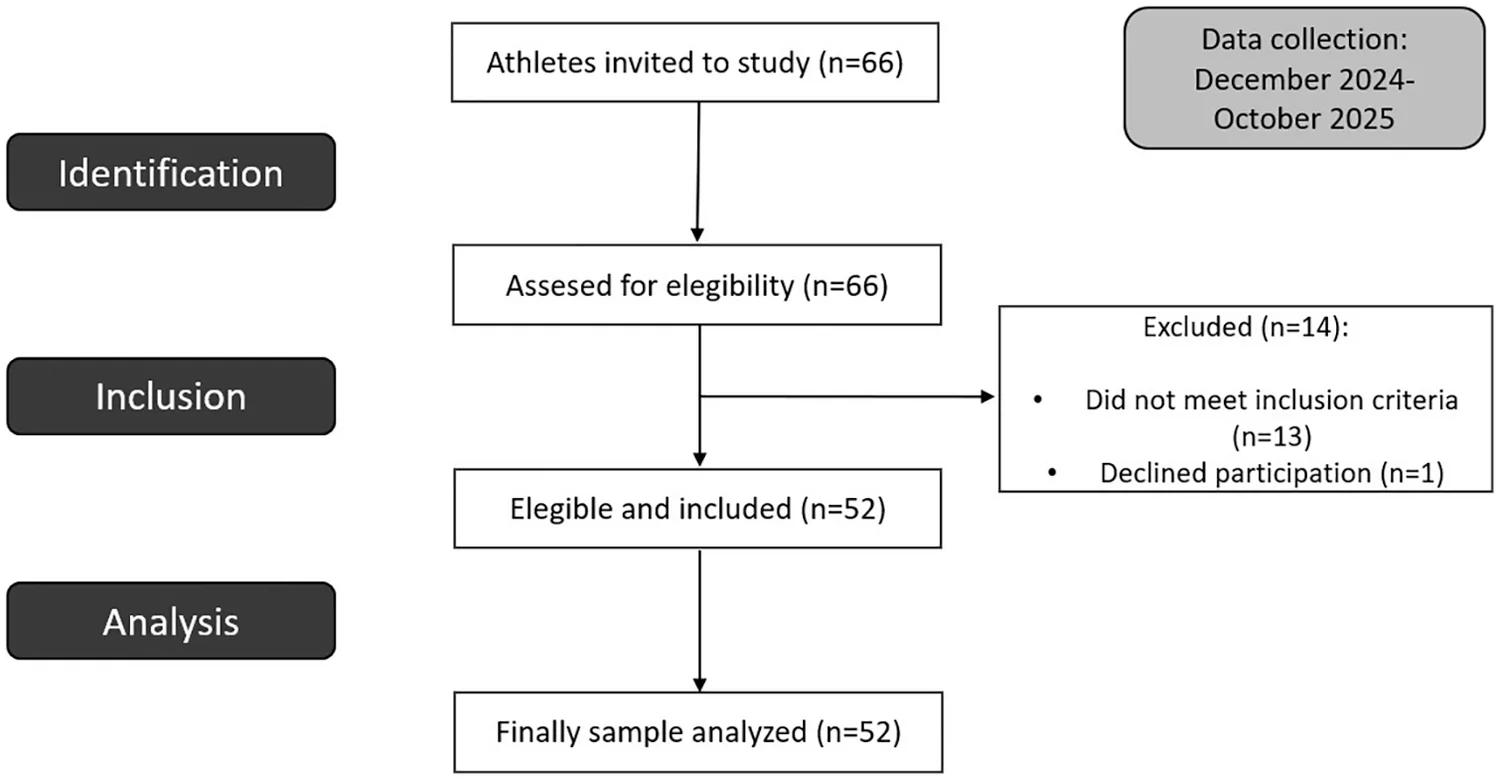
Flowchart depicting the selection process of internship centers and participants included in the final analysis.

### Outcomes

#### Maximum isometric strength of the external and internal rotators of the shoulder joint complex

Shoulder strength was assessed using the Active Force 2 handheld dynamometer (Activbody Inc., San Diego, CA, USA), a device previously used for the assessment of shoulder strength (21). The testing procedure was based on previously described handheld dynamometry methods (22), with modifications to the shoulder testing position and trial protocol to better reflect the functional demands of overhead athletes. Handheld dynamometry has demonstrated good-to-excellent reliability for measuring shoulder internal and external rotator strength when standardized testing procedures are used. All assessments were performed under resting conditions, without a prior warm-up.

Prior to data collection, participants received a clear explanation of the procedure and were allowed time to familiarize themselves with the equipment. Participants then performed a standardized 3-minute warm-up followed by two submaximal practice trials to familiarize themselves with the testing procedure and ensure consistent execution before maximal strength assessment. This approach was consistent with previous research supporting standardized familiarization and testing procedures for handheld dynamometry (21, 23). Strength values were recorded in kilograms (kg).

For the measurements, participants lay supine on a stretcher, with their torso stabilized using hook-and-loop (Velcro®) straps to minimize compensatory movements. Shoulder internal and external rotation strength was assessed with the shoulder positioned at 90° of abduction and the elbow flexed to 90°. This standardized position has also been used for assessing maximal isometric shoulder internal and external rotation strength using a handheld dynamometer in overhead athletes (23) (Figure 2).

**Figure 2:**
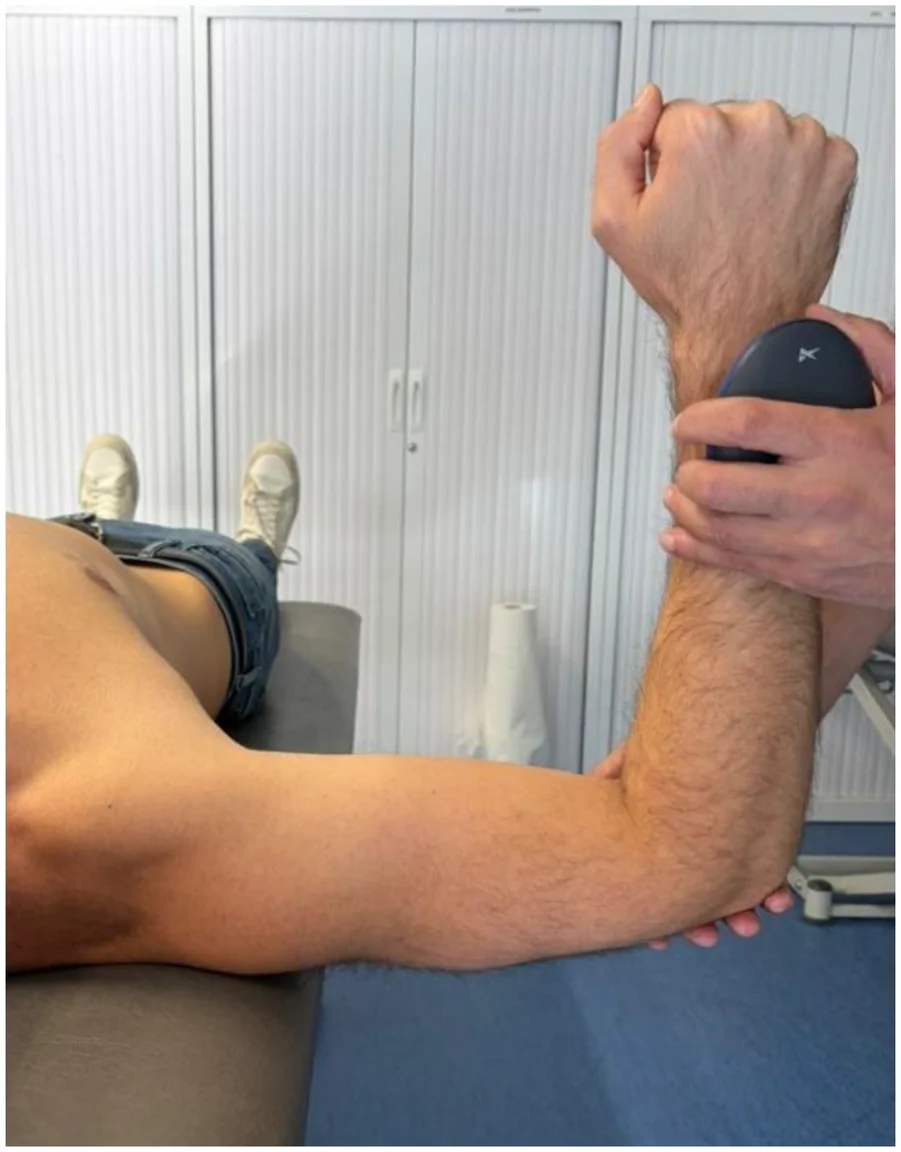
Testing position for isometric measurement of shoulder rotator strength.

The dynamometer was placed transversely on the distal forearm, immediately proximal to the wrist joint. During testing, the examiner stood facing the participant at the side of the examination table and applied counterforce through the handheld dynamometer positioned immediately proximal to the wrist. Trunk stabilization was achieved using hook-and-loop straps, while the examiner maintained the testing position throughout each contraction to ensure a purely isometric effort and minimize compensatory movements. Three maximal voluntary isometric contractions of 5 s each were performed for both shoulder internal and external rotation, with a 30-second rest period between trials. This approach was selected to reduce the influence of occasional submaximal efforts and to obtain a representative measure of maximal voluntary strength.

Throughout testing, the physiotherapist maintained consistent pressure against the dynamometer to match the participant's effort while ensuring that the limb remained static. Standardized verbal encouragement was provided, and participants were instructed to avoid performing the Valsalva maneuver. All assessments were performed by the same physiotherapist, with more than five years of experience in musculoskeletal strength assessment and specific training in handheld dynamometry. To minimize measurement variability, standardized participant positioning, trunk stabilization using hook-and-loop straps, and consistent examiner positioning were maintained throughout all assessments.

Peak force was automatically recorded by the device, and the highest value obtained across the three trials was used for statistical analysis because the purpose of the assessment was to quantify maximal voluntary isometric strength rather than the average of repeated trials.

#### Internal-to-external rotator strength ratio

The internal-to-external rotator strength ratio (IR/ER ratio) was calculated by dividing maximal isometric internal rotation strength by maximal isometric external rotation strength for each shoulder. As the IR/ER ratio is influenced by the testing protocol and shoulder position, its interpretation was limited to comparisons performed under identical assessment conditions (24).

#### Scapular position assessment using the PALM® device

Static scapular position was assessed using the PALM® (PALpation Meter; Performance Attainment Associates, Roseville, MN, USA), following the methodology described by da Costa et al. (25). The PALM® was selected because it provides an objective, inexpensive, and clinically applicable method for quantifying static scapular position under standardized conditions. Although static scapular measurements do not capture dynamic scapular kinematics, they remain useful for describing scapular position and identifying potential side-to-side or between-group differences in observational studies. Previous studies have demonstrated good to excellent reliability for PALM® measurements of static scapular position, with intra-rater ICCs ranging from 0.81 to 0.89 and inter-rater ICCs ranging from 0.67 to 0.89 (25).

The PALM® device was positioned with one caliper tip placed on the inferior angle of the scapula and the other on the spinous process located at the same horizontal level as the inferior scapular angle, following the anatomical landmarking procedure described by da Costa et al. (2010). The same procedure was used for all participants and for both testing positions (Figure 3). The difference between both measurements was calculated to quantify the change in scapular position during arm elevation. Bilateral comparisons were performed to identify potential asymmetries in scapular position and scapular displacement between shoulders.

**Figure 3:**
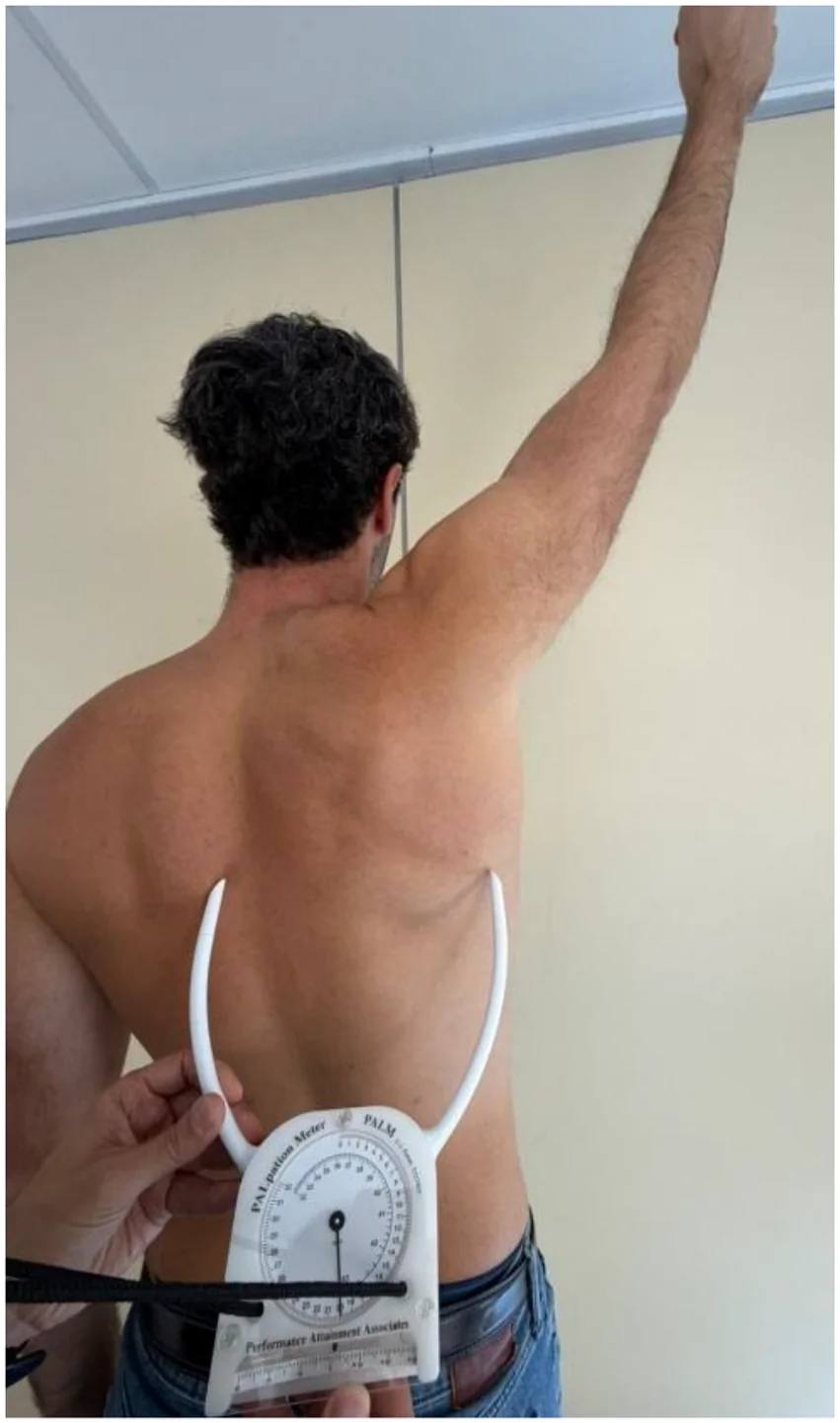
Assessment of static scapular position using the PALM® device. One caliper tip was positioned over the inferior angle of the scapula, while the opposite tip was placed over the thoracic spinous process located at the same horizontal level.

#### Ultrasound measurement procedure

Supraspinatus tendon thickness was assessed using a Mindray DC-70 ultrasound system (Mindray, Shenzhen, China) equipped with a high-frequency linear transducer (7-12 MHz), which was positioned perpendicular to the tendon to avoid image distortion and ensure accurate measurements.

All participants were examined in a seated position with the tested arm placed in the modified Crass position (that is, with the hand resting on the lower back. This posture allows for optimal exposure of the supraspinatus tendon beneath the acromion) (26).

For the supraspinatus tendon, the transducer was placed longitudinally over the supraspinous fossa, aligning the image with the tendon's major axis. Thickness was measured from the articular surface to the subacromial bursa (Figure 4). To ensure measurement reliability, three consecutive measurements of tendon thickness were taken at the most homogeneous and proximal region of the tendon. The mean value of the three measurements was used for analysis. The ultrasound protocol was selected because previous studies have demonstrated excellent reliability for measuring supraspinatus tendon thickness. Reported intra-rater and inter-rater reliability coefficients are generally excellent (ICC > 0.90), with minimal detectable change (MDC) values ranging from approximately 0.6 to 1.3 mm, depending on the examiner's experience and the measurement protocol (26).

**Figure 4:**
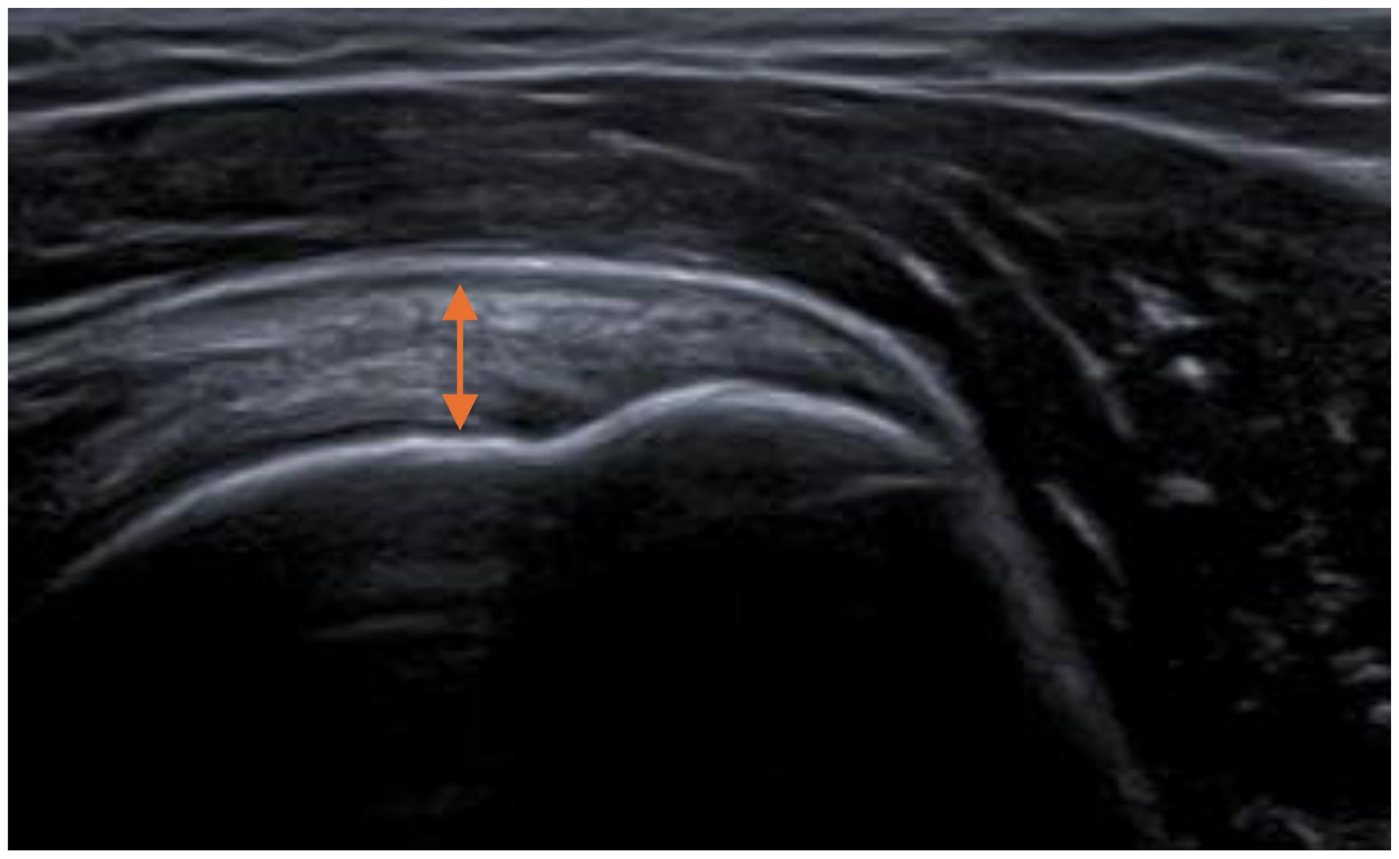
Ultrasound measurement of supraspinatus tendon thickness in longitudinal view.

### Methodological considerations

All measurements were performed by the same experienced physiotherapist using standardized protocols to ensure consistency and reduce inter-examiner variability. Prior to data collection, the assessor was trained in the use of all instruments, including the handheld dynamometer, PALM® device, and ultrasound procedures.

Due to the nature of the study, blinding of the assessor was not feasible, as all measurements required direct interaction with the participants. However, standardized positioning, clear procedural protocols, and consistent measurement conditions were applied to minimize potential measurement bias.

To reduce the influence of confounding factors, participants were selected based on similar inclusion criteria, including age range, training frequency, competitive level, and absence of recent shoulder injury. Although variables such as playing position, training load variability, and previous injury history were not fully controlled, all participants were actively competing and followed comparable training routines.

All participants underwent the assessments in the same standardized order to ensure procedural consistency. Anthropometric measurements were performed first, followed by scapular position assessment using the PALM®, ultrasound examination of the supraspinatus tendon, and finally shoulder strength assessment using handheld dynamometry. Both shoulders were evaluated in the same sequence for all participants, beginning with the dominant limb followed by the non-dominant limb. No randomization of the assessment order was performed.

### Statistical analysis

Data analyses were performed using Jamovi (Version 2.6.44; The jamovi project, 2024). Mean ± SD was used for the description of quantitative variables. The normality of the data was assessed using the Shapiro–Wilk test. Differences between groups were analyzed using Student's t-test for normally distributed variables and the Mann–Whitney U test for non-normally distributed variables. The level of significance was set at ≤0.05.

Effect sizes (Cohen's d for normally distributed variables; rank-biserial correlation for non-normally distributed variables) and their 95% confidence intervals (95% CI) were calculated for all comparisons. For non-parametric comparisons, 95% CI for the rank-biserial correlation were estimated using the normal approximation method (Kerby, 2014), as bootstrap or exact confidence intervals were not available in the statistical software used.

Given the significant differences in height and weight between male and female athletes, multiple linear regression models (with sex, height, and weights predictors) were performed for the four variables showing significant sex differences in the univariate analysis (internal rotation strength, internal/external rotation ratio, Palm test in flexion, and Scapular displacement). Since there was evidence of heteroscedasticity across sex groups, inferential statistics were computed using heteroscedasticity-consistent (robust) standard errors. No relevant collinearity was found among predictors (all VIF < 2). To account for multiple comparisons across the five outcome variables, *p*-values for the sex effect were adjusted using the Holm-Bonferroni correction.

## Results

A total of 52 water polo players were recruited, 26 males aged 18.46 ± 3.04 and 26 females aged 18.73 ± 2.98. Among them, 44 (84.6%) had right-arm dominance, 22 participants of each sex. Table 1 shows the demographic data of the sample.

**Table 1:** Participant demographic data. Mean ± standard deviation.

Data	Male (*n* = 26)	Female (*n* = 26)	*p*-value
Age, years	18.73 ± 2.98	18.46 ± 3.04	0.678^a^
Weight, kg	72.73 ± 8.13	67.53 ± 4.02	**0**.**001**^b^
Height, m	1.77 ± 0.07	1.69 ± 0.01	**<0**.**001**^b^
BMI (kg/m^2^)	23.36 ± 2.48	23.19 ± 2.87	0.855^a^
Weekly training volume, days	4.96 ± 0.19	4.88 ± 0.32	0.312^a^

Bold indicates statistically significant differences.

a Mann–Whitney U test.

b Student's t-test.

Values of biomechanical and anatomic variables, as well as between groups univariate analyses, are shown by gender in Table 2 and by arm dominance in Table 3.

**Table 2:** Shoulder biomechanical and anatomic measurements by gender.

Variable	Male (*n* = 26)	Female (*n* = 26)	*p*-value	Cohen's d (95% CI)	Magnitude
Internal rotation strength (kg)	15.94 ± 4.56	13.32 ± 2.10	**0**.**011**^a^	0.74 (0.17, 1.29)	Moderate–large
External rotation strength (kg)	13.17 ± 2.98	11.26 ± 3.75	0.052^a^	0.32 (−0.002, 0.63)	Moderate
Internal/external rotation ratio	1.27 ± 0.23	1.11 ± 0.12	**0**.**004**^b^	0.87 (0.272, 1.409)	Large
Palm test rest (cm)	10.64 ± 1.47	10.84 ± 1.47	0.920^a^	−0.00 (0.32, 0.32)	Trivial
Palm test in flexion (cm)	16.50 ± 1.49	17.73 ± 1.60	**0**.**006**^b^	−0.79 (−1.36, −0.23)	Moderate–large
Scapular displacement (cm)	5.85 ± 1.87	7.08 ± 0.87	**0**.**004**^b^	−0.85 (−1.41, −0.27)	Large
Supraspinatus tendon thickness (cm)	0.63 ± 0.10	0.61 ± 0.09	0.384^a^	0.14 (−0.18, 0.46)	Small

Bold indicates statistically significant differences.

a Mann–Whitney U test.

b Student's t-test.

**Table 3:** Shoulder biomechanical and anatomic measurements by arm dominance.

Variable	Right (*n* = 44)	Left (*n* = 8)	*p*-value	Cohen's d	Magnitude
Internal rotation strength	14.78 ± 3.96	13.86 ± 2.40	0.621^a^	0.11 (−0.33, 0.55)	Small
External rotation strength	12.04 ± 3.67	13.43 ± 2.14	0.361^a^	−0.21 (−0.65, 0.23)	Small–moderate
Internal/external ratio	1.20 ± 0.21	1.14 ± 0.13	0.393^b^	0.31 (−0.426, 1.09)	Small
Palm test rest	10.57 ± 1.51	11.09 ± 1.18	0.483^a^	−0.16 (−0.60, 0.28)	Small
Palm test in flexion	17.18 ± 1.68	16.76 ± 1.55	0.518^b^	0.25 (−0.51, 1.00)	Small
Scapular displacement	6.61 ± 1.49	5.68 ± 1.89	0.125^b^	0.60 (−0.17, 1.36)	Moderate
Supraspinatus thickness	0.63 ± 0.11	0.63 ± 0.03	0.760^a^	0.07 (−0.51, 0.37)	Trivial

a Mann–Whitney U test.

b Student's t-test.

In univariate analyses, males exhibited significantly greater shoulder internal rotation strength compared to females. Although external rotation (ER) strength was also higher in males, this difference did not reach statistical significance (*p* = 0.052). However, the IR/ER ratio was higher in men, indicating a greater imbalance between rotator muscle groups.

With respect to scapular displacement assessed using the PALM® device, no significant differences were observed at rest between sexes. However, during maximal shoulder flexion, women demonstrated significantly greater scapular displacement, resulting in a higher total displacement compared to men.

Effect size analysis revealed moderate-to-large differences in internal rotation strength (d = 0.74) and large differences in the IR/ER ratio (d = 0.87) and scapular displacement (d = 0.85), indicating clinically relevant sex-related differences.

Finally, no significant differences were found in supraspinatus tendon thickness between male and female participants, with only a small effect size observed (d = 0.21).

No significant differences were observed between right- and left-dominant athletes for any of the variables analyzed (*p* > 0.05). Effect size analysis revealed predominantly small or trivial differences across variables, with only a moderate effect observed for scapular displacement (d = 0.60), which did not reach statistical significance.

Multiple linear regression models were performed for the four variables showing sex differences in the univariate analyses, with sex, height, and body mass included as predictors. Adjustment for anthropometric variables (height and body mass) using multiple linear regression models is shown in Table 4.

**Table 4:** Multiple linear regression models (sex, height, and weight) for variables showing significant sex differences in univariate analysis.

Variable	Predictor	B	95% CI (B)	*β*	95% CI (*β*)	p
Internal rotation strength	Sex (M–F)	1.245	[−1.074, 3.565]	0.331	[−0.286, 0.948]	0.331
	Height	3.233	[−13.247, 19.713]	0.065	[−0.265, 0.395]	0.735
	Body mass	0.176	[0.012, 0.340]	0.332	[0.023, 0.641]	**0**.**014**
Internal/external rotation ratio	Sex (M–F)	0.119	[−0.007, 0.245]	0.600	[−0.033, 1.232]	0.177
	Height	0.066	[−0.827, 0.959]	0.025	[−0.314, 0.364]	0.887
	Body mass	0.005	[−0.004, 0.014]	0.179	[−0.137, 0.496]	0.373
PALM test in flexion	Sex (M–F)	−1.368	[−2.411, −0.324]	−0.826	[−1.456, −0.196]	0.057
	Height	−3.219	[−10.635, 4.196]	−0.146	[−0.483, 0.191]	0.527
	Body mass	0.064	[−0.009, 0.138]	0.275	[−0.040, 0.590]	0.170
Scapular displacement	Sex (M–F)	−1.342	[−2.351, −0.333]	−0.850	[−1.490, −0.211]	**0**.**005**
	Height	2.750	[−4.419, 9.919]	0.131	[−0.211, 0.473]	0.513
	Body mass	−0.019	[−0.091, 0.052]	−0.087	[−0.407, 0.233]	0.618

Model R^2^: Internal rotation strength = 0.226; Internal/external rotation ratio = 0.185; Palm test in flexion = 0.193; Scapular displacement = 0.169. All VIF < 2 (no relevant multicollinearity). Inferential tests and *p*-values were adjusted for heteroscedasticity (robust standard errors). B, unstandardized regression coefficient; β, standardized regression coefficient.

The bold values correspond to statistically significant associations (*p* < 0.05).

After adjustment, sex was no longer a significant predictor of internal rotation strength (*β* = 0.331, *p* = 0.331), IR/ER ratio (*β* = 0.600, *p* = 0.177), and nearly (but not significant) of Palm test in flexion (*β* = −0.826, *p* = 0.057), indicating these crude differences were largely attributable to anthropometric variation. Weight was an independent significant predictor of internal rotation strength (*p* = 0.014). In contrast, sex remained a significant predictor of Scapular displacement after adjustment (*β* = −0.850, *p* = 0.005), and this result remained significant after Holm-Bonferroni correction for multiple comparisons across the four outcomes (adjusted *p* = 0.020), suggesting a sex-specific biomechanical characteristic independent of body size.

## Discussion

The present study aimed to provide a sport-specific characterization of shoulder function in male and female water polo players, integrating strength, internal-to-external rotator strength ratio, scapular displacement, and tendon morphology. Although univariate analyses showed sex-related differences in shoulder strength and scapular displacement, adjustment for height and body mass demonstrated that only scapular displacement remained independently associated with sex. These findings suggest that anthropometric characteristics largely explain strength differences, whereas scapular mechanics may represent a genuine sex-specific adaptation in competitive water polo players.

Based on previous evidence from overhead sports and the limited data available in water polo, we hypothesized that male and female players would exhibit different functional and structural shoulder profiles. This hypothesis was partially supported, as sex-related differences were observed in scapular displacement, whereas differences in shoulder strength and internal-to-external rotation strength ratio were largely explained by anthropometric characteristics, and no differences were found in supraspinatus tendon thickness. These findings suggest that sex-related differences in shoulder adaptations among water polo players may be more evident in dynamic biomechanical characteristics than in absolute strength or tendon morphology.

Because of the exploratory cross-sectional design, these findings should not be interpreted as evidence of causality or direct predictors of injury risk. Nevertheless, they provide new information regarding shoulder function in a sport for which sex-specific evidence remains scarce.

Regarding shoulder strength, male players demonstrated greater internal rotation strength and a higher IR/ER ratio in the univariate analysis. Similar findings have been reported in overhead athletes and are generally attributed to greater lean muscle mass, hormonal differences, and higher absolute training loads (27).

However, after adjustment for height and body mass, neither internal rotation strength nor the IR/ER ratio remained significantly associated with sex. This indicates that these differences were largely explained by anthropometric characteristics rather than sex itself. Consequently, caution is warranted when interpreting absolute strength differences between male and female athletes, as body size appears to be an important determinant of shoulder strength.

This finding is consistent with previous methodological evidence showing that absolute muscle strength is strongly influenced by body size and that comparisons between groups should be interpreted cautiously unless anthropometric differences are taken into account (28). In contrast, the persistence of sex differences in scapular displacement after anthropometric adjustment suggests that factors beyond body size, such as neuromuscular control strategies or tissue mechanical properties, may contribute to sex-specific shoulder adaptations in water polo players.

Although an elevated IR/ER ratio has been associated with shoulder dysfunction in overhead athletes (29), the present findings do not support sex as an independent determinant of this ratio in competitive water polo players. Therefore, rotator cuff conditioning should be individualized according to each athlete's functional profile rather than sex alone.

The most consistent sex-related finding in the present study concerned scapular displacement. Scapular dyskinesis is common in overhead athletes and has been associated with altered neuromuscular control and shoulder disorders (30, 31). However, evidence in water polo remains limited (4).

Female athletes exhibited greater scapular displacement during shoulder elevation, whereas no differences were observed at rest. Importantly, this association remained significant after adjustment for anthropometric variables and after Holm–Bonferroni correction, suggesting that it reflects a true sex-related biomechanical characteristic rather than differences in body size.

This finding is consistent with studies suggesting that women generally present greater joint laxity, lower passive stiffness, and different neuromuscular control strategies, all of which may influence scapular motion (32). Greater displacement should not necessarily be interpreted as pathological; rather, it may represent a functional adaptation that warrants further longitudinal investigation to determine whether it is associated with shoulder pain or injury.

Although anthropometric adjustment eliminated the observed sex differences in shoulder strength, it is important to consider the specific competitive demands of water polo. Men and women compete under essentially the same playing conditions, including identical pool dimensions and match duration, while using balls of the same weight despite slight differences in ball size. Consequently, female players may experience a relatively greater mechanical burden on the shoulder during repetitive overhead actions. Although this hypothesis was not evaluated in the present study, it may partly explain why greater scapular displacement was observed in female athletes and deserves further investigation using biomechanical and longitudinal study designs.

Previous water polo studies have largely focused on male elite athletes (31, 33), limiting comparisons with the present mixed-sex sample. Therefore, the current study contributes novel evidence by identifying sex-related differences in dynamic scapular behaviour in competitive male and female water polo players.

In contrast to the functional differences observed in scapular displacement, no significant sex differences were identified in supraspinatus tendon thickness, supporting previous findings suggesting that tendon morphology is primarily influenced by cumulative mechanical loading and training exposure rather than sex (18, 34).

These results indicate that functional differences between sexes are not accompanied by detectable structural differences in tendon thickness, although ultrasound morphology alone cannot determine tendon health or tissue quality.

Finally, no significant differences were found according to arm dominance. Although this result should be interpreted cautiously because of the limited number of left-handed players, it agrees with previous reports in aquatic sports suggesting that repetitive bilateral training may reduce functional asymmetries (Aginsky et al., 2016; Batalha et al., 2014).

### Clinical implications

The present findings support an individualized approach to shoulder assessment in competitive water polo players rather than relying solely on sex-based differences. Although unadjusted analyses showed greater shoulder strength in male athletes, these differences were largely explained by anthropometric characteristics after adjustment.

Therefore, strength assessment and conditioning programs should primarily consider each athlete's individual functional profile rather than sex alone. In contrast, the greater scapular displacement observed in female players suggests that scapular control and neuromuscular training may deserve particular attention when designing preventive and conditioning programs, although prospective studies are needed to determine its relationship with shoulder injury risk.

## Limitations

This study has several limitations that should be acknowledged. First, the relatively small sample size and the use of convenience sampling may limit the generalizability of the findings to broader populations of water polo players. No formal *a priori* sample size calculation was performed because this was an exploratory cross-sectional study based on the recruitment of all eligible athletes from two federated clubs. Although the final sample was considered adequate to detect large between-group differences, the study may have been underpowered to identify smaller effects. Therefore, the findings should be considered preliminary and interpreted cautiously, and the study may be regarded as an exploratory investigation that provides initial evidence on sex-specific shoulder profiles and generates hypotheses for future research. Furthermore, although regression models were adjusted for height and body mass, the modest sample size may have reduced the precision of the adjusted estimates.

Second, although all participants were field players and were matched according to sex and competitive level, playing position (e.g., center forward, driver, winger, point, or center back) was not systematically recorded and therefore could not be controlled for in the analyses. As different playing positions may involve different throwing volumes and biomechanical demands, this factor may have influenced the observed shoulder characteristics. In addition, other potentially relevant factors, including years of competitive experience, training load, and previous shoulder injury history, were not fully controlled. Future studies should account for these variables to better understand their contribution to shoulder adaptations in water polo players.

Third, the cross-sectional design precludes the establishment of causal relationships between shoulder functional characteristics and injury risk. Consequently, it cannot be determined whether the observed differences, particularly the greater scapular displacement in female athletes, represent adaptive sport-specific characteristics or potential risk factors for future shoulder disorders. Longitudinal studies are required to clarify these relationships.

Fourth, scapular assessment was performed using the PALM®, a reliable and clinically applicable instrument for measuring static and functional scapular position. Nevertheless, this method does not provide three-dimensional kinematic analysis and therefore cannot fully characterize dynamic scapular motion during sport-specific activities such as throwing. Regarding limb dominance, no significant differences were observed between right- and left-dominant athletes. However, this finding should be interpreted with caution, as the predominance of right-handed participants (*n* = 44) may have limited the statistical power to detect potential differences related to limb dominance.

Finally, all measurements were performed by a single experienced examiner using standardized protocols. Although this approach minimized inter-examiner variability and ensured methodological consistency, the lack of assessor blinding may have introduced measurement bias. Furthermore, the findings are limited to young competitive water polo players competing at regional and national levels. Therefore, caution is warranted when extrapolating these results to younger athletes, recreational players, elite international competitors, or other overhead sports.

## Conclusions

Male players demonstrated greater shoulder internal rotator strength and a higher external rotator strength ratio in unadjusted analyses; however, these differences were largely explained by anthropometric characteristics after adjustment for height and body mass. In contrast, greater scapular displacement in female athletes remained independently associated with sex, suggesting a genuine sex-specific biomechanical adaptation. No sex-related differences were observed in supraspinatus tendon thickness or according to arm dominance.

These findings indicate that shoulder assessment in competitive water polo should prioritize individual functional characteristics rather than absolute strength differences alone. Future longitudinal studies are needed to determine whether the greater scapular displacement observed in female players is associated with shoulder pain, performance, or injury risk.

## Data Availability

The raw data supporting the conclusions of this article will be made available by the authors, without undue reservation.
